# Glycolic Acid-Guided Intelligent Neurovascular Imaging: A Cross-Scale Platform for Real-Time Neuroprotection and Adaptive Stroke Imaging

**DOI:** 10.3390/jcm15051851

**Published:** 2026-02-28

**Authors:** Krzysztof Malczewski, Ryszard Kozera, Zdzislaw Gajewski, Maria Sady

**Affiliations:** 1 Institute of Information Technology, Warsaw University of Life Sciences, Nowoursynowska St. 159, Building 34, 02-776 Warsaw, Poland; 2 Center of Translational Medicine, Warsaw University of Life Sciences, Nowoursynowska St. 100, 02-797 Warsaw, Poland; zdzislaw_gajewski@sggw.edu.pl (Z.G.);

**Keywords:** acute ischemic stroke, MR–PET hybrid imaging, compressed sensing, persistent homology, topology-preserving reconstruction, neuroprotection, glycolic acid, targeted micro-radioembolization, theranostics

## Abstract

**Introduction:** Acute ischemic stroke demands interventions that restore perfusion and protect neurons within a narrow therapeutic window. We propose a unified theranostic platform that couples adaptive imaging, topology-aware decision-making, and immediate neuroprotective and micro-dosimetric intervention. **Methods:** The platform integrates three components. First, a topology-preserving MR–PET engine employs adaptive Poisson-disc sampling, partial Fourier constraints, and structured Hankel low-rank priors in a closed loop. Persistent-homology metrics quantify vascular graph uncertainty and guide subsequent k-space and PET projections, reducing acquisition time while preserving collateral topology. Second, immediate post-reperfusion delivery of glycolic acid attenuates glutamate-driven calcium influx and stabilizes mitochondrial function. Third, trace doses of sol–gel-derived, neutron-activated ^90^Y_2_O_3_ microspheres provide sharply confined beta irradiation for micro-scale metabolic modulation. **Results:** In a porcine stroke model replicating the human recanalization workflow, the imaging engine maintained vascular Betti-number invariants within three percent of fully sampled reference scans while reducing acquisition time by nearly half. Glycolic acid reduced glutamate-induced intracellular calcium rise by approximately sixty percent in vitro and decreased infarct volume by thirty-eight percent in vivo. Micro-dosimetry confirmed a mean perivascular beta dose of twenty-eight grays, and histology demonstrated a forty-two percent increase in NeuN-positive neuronal survival compared with standard recanalization. **Conclusions:** These results demonstrate that intelligent compressed-sensing MR–PET, targeted micro-radioembolization, and glycolic acid neuroprotection can act synergistically to bridge diagnostic imaging and immediate intervention. By coupling imaging, decision-making, and therapy in a closed-loop manner and elevating topological fidelity from a reconstruction byproduct to a control variable, the proposed platform reframes MR–PET from passive diagnostics into an active, decision-driven theranostic system and establishes a foundation for future human trials.

## 1. Introduction

Stroke remains one of the most formidable challenges to modern medicine, consistently ranking among the leading causes of death and long-term disability. According to the Global Burden of Disease 2019 analysis, ischemic stroke alone is responsible for more than 5.5 million deaths and over 116 million disability-adjusted life years annually worldwide [1]. Rapid recanalization through intravenous thrombolysis or mechanical thrombectomy has transformed acute care, yet even in well-organized stroke networks, only a minority of patients receive these treatments in time and, crucially, successful reopening of the occluded artery does not itself prevent the cascade of excitotoxic and metabolic injury that follows ischemia–reperfusion [2]. The unmet need is therefore for an integrated approach that combines fast and accurate imaging with immediate, biologically effective neuroprotection and quantifiable micro-therapeutic modulation of the ischemic tissue environment.

Recent advances in three complementary research areas now make such an approach conceivable. First, magnetic resonance imaging (MRI) and positron emission tomography (PET) remain indispensable for characterizing the cerebral vasculature, collateral perfusion and tissue metabolism, but conventional protocols require long acquisitions and fixed sampling trajectories. These constraints delay clinical decision-making and can obscure the very distal vascular features most critical for therapy. Compressed sensing (CS) demonstrated that images that are sparse in an appropriate transform domain can be reconstructed from far fewer samples than prescribed by the Nyquist criterion [3]. Subsequent developments have applied adaptive Poisson-disc sampling, partial Fourier constraints and structured Hankel low-rank priors to dramatically accelerate MR and hybrid MR–PET imaging while preserving fine vascular detail [4]. Yet most current implementations still rely on static sampling masks and do not exploit the evolving information revealed during reconstruction. A truly intelligent imaging engine should instead quantify reconstruction uncertainty in real time and adaptively steer acquisition so that each newly acquired datum maximally improves the clinical relevance of the images.

Secondly, the search for effective neuroprotection has led to an unexpected discovery in the biology of desiccation-tolerant *Caenorhabditis elegans*. These nematodes survive extreme anoxic stress through a metabolic stop–start cycle and accumulate glycolic acid (GA) via the glyoxalase DJ-1 pathway. Translating this observation to mammalian systems revealed that GA can markedly reduce glutamate-dependent intracellular calcium influx, preserve mitochondrial integrity and protect neurons from ischemia–reperfusion injury. Importantly, the beneficial effect was reproduced not only in rodent models but also in large-animal stroke experiments, providing a rare example of a naturally occurring metabolite with robust neuroprotective activity in a setting that mirrors human disease [5]. Despite its promise, GA has not yet been incorporated into an integrated therapeutic-imaging workflow capable of monitoring its delivery and linking its molecular effects to tissue outcome in real time.

Thirdly, advances in targeted radionuclide therapy have produced sol–gel-synthesized, neutron-activated yttrium oxide (^90^Y_2_O_3_) microspheres with diameters of 20–100 µm and radionuclide purities exceeding 99.99%. These microspheres emit high-energy beta particles and can deliver spatially confined radiation with predictable micro-dosimetry, properties that are already exploited in hepatic radioembolization but have not yet been applied to the neurovascular domain [6]. Controlled micro-radioembolization within reperfused brain tissue could provide a means to modulate the peri-infarct microenvironment, influence inflammatory responses or complement metabolic therapies such as GA without damaging surrounding structures.

Progress towards clinical translation, however, is often hindered by the limitations of small-animal models. Rodent brains differ markedly from those of humans in cortical folding, white-to-gray matter ratio and collateral circulation, contributing to the repeated failure of neuroprotective strategies that were successful in rodents but are ineffective in patients. By contrast, the gyrencephalic porcine brain closely resembles human cerebrovascular anatomy and allows endovascular induction of stroke and subsequent recanalization with clinical catheters under continuous MRI guidance [7]. Such large-animal models offer an essential bridge for evaluating not only neuroprotective agents but also complex interventional and imaging technologies in conditions that replicate human workflow and instrumentation.

These parallel but until now separate advances highlight both an opportunity and a critical scientific gap. Intelligent compressed-sensing MR–PET imaging can dramatically accelerate acquisition and preserve the topology of the cerebral vasculature, but it has not been linked to real-time therapeutic interventions. Glycolic acid provides compelling evidence of molecular neuroprotection in large animals yet lacks an integrated imaging framework that can guide delivery and correlate biochemical effects with evolving perfusion and metabolism. Yttrium-90 microspheres enable precise micro-dosimetry and potential metabolic modulation, but they too have never been combined with adaptive imaging or neuroprotective pharmacology. No existing platform unites these three advances into a single, closed-loop system capable of delivering therapy, monitoring its effects and quantifying tissue outcome within the narrow time window of acute stroke care.

The present study addresses this gap by introducing a hybrid MR–PET theranostic platform that brings together these strands of research. The system integrates topology-aware, uncertainty-driven compressed sensing to adaptively accelerate MR–PET acquisition without loss of collateral vessel fidelity; intra-arterial administration of glycolic acid at the moment of reperfusion to counteract calcium-mediated excitotoxicity and stabilize mitochondrial function; and trace-dose micro-radioembolization with sol–gel-derived ^90^Y_2_O_3_ microspheres to provide spatially confined beta irradiation and quantitative micro-dosimetry. Validation in a porcine model of endovascularly induced middle cerebral artery occlusion with subsequent rtPA recanalization demonstrates that this cross-scale approach links macrovascular topology, meso-scale perfusion-dosimetry and micro-scale neuronal survival. By uniting advanced digital signal processing, molecular neuroprotection and targeted radionuclide therapy in a single time-critical workflow, this work establishes a new paradigm for precision neurovascular medicine and provides a credible blueprint for translation to first-in-human trials.

Acute ischemic stroke management is fundamentally constrained by time-to-decision and uncertainty regarding tissue viability and collateral circulation. Large-animal models are increasingly recognized as critical for bridging the translational gap between rodent studies and human stroke, as vascular geometry, brain size, and hemodynamic scaling substantially affect infarct evolution and treatment response [8]. The present work addresses both challenges by combining rapid, topology-preserving MR–PET imaging with real-time therapeutic guidance. By reducing acquisition time to approximately one minute while preserving vascular graph fidelity, the proposed workflow has the potential to accelerate reperfusion decisions, refine patient selection for adjunctive neuroprotective therapies, and enable image-guided micro-interventions within the same procedural window.

## 2. Related Work and Research Gap

The scientific landscape relevant to this study spans three rapidly developing fields: adaptive compressed-sensing MR–PET imaging, molecular neuroprotection during ischemia–reperfusion, and targeted radionuclide micro-therapies. Each area has progressed considerably over the past decade, yet each exhibits limitations that motivate the integrated approach proposed here.

Magnetic resonance imaging (MRI) and positron emission tomography (PET) remain indispensable for detecting large-vessel occlusion, assessing collateral circulation and characterizing the metabolic state of the ischemic penumbra. Hybrid PET/MR reconstruction has previously been explored using combinations of compressed sensing and super-resolution to mitigate the trade-off between acquisition speed and spatial resolution [9], but these approaches do not explicitly account for topological fidelity or closed-loop acquisition control. Conventional MR and hybrid MR–PET protocols, however, require lengthy acquisitions and employ fixed sampling strategies that do not adapt to the evolving diagnostic information during a scan. The seminal work of Lustig and colleagues demonstrated that compressed sensing (CS) could recover images that were sparse in an appropriate transform domain from dramatically fewer measurements than dictated by the Nyquist criterion [3]. Since that breakthrough, numerous refinements have been introduced to accelerate vascular imaging, including adaptive Poisson-disc sampling for incoherent k-space coverage, partial Fourier constraints to reduce echo-train length, and structured Hankel low-rank priors to stabilize high-frequency detail [4]. Despite these advances, most CS-based clinical protocols still employ static masks determined a priori and are not informed by reconstruction uncertainty or downstream clinical tasks. Moreover, current super-resolution and deep-learning methods frequently optimize for pixel-wise or perceptual fidelity rather than preservation of vascular topology, leaving collateral networks vulnerable to artifacts and potentially misleading in the context of endovascular therapy planning.

Parallel to these developments, translational neuroscience has sought pharmacological strategies to protect neurons from ischemia–reperfusion injury. Excitotoxic influx of calcium ions, triggered by excessive glutamate receptor activation, is recognized as a central mechanism of delayed neuronal death after reperfusion. While hundreds of agents have demonstrated neuroprotection in small-animal models, clinical translation has repeatedly failed. A striking recent discovery identified glycolic acid (GA) as an endogenous metabolite capable of mitigating calcium-mediated excitotoxicity and preserving mitochondrial integrity. Originally observed in desiccation-tolerant *Caenorhabditis elegans*, GA was shown to protect mammalian neurons in vitro and to reduce infarct volume in both rodent and large-animal stroke models [5]. Although these findings establish GA as a credible candidate for clinical neuroprotection, no existing imaging framework provides real-time guidance of its delivery or quantitative correlation between GA distribution, perfusion dynamics and ultimate tissue survival.

A third relevant field is targeted radionuclide therapy. Sol–gel synthesis and neutron activation have enabled the production of yttrium oxide (^90^Y_2_O_3_) microspheres with tightly controlled diameters (20–100 µm) and radionuclide purity exceeding 99.99%. These microspheres emit high-energy beta particles and are already used for hepatic radioembolization, delivering predictable micro-dosimetry and sharply confined radiation [6]. Their potential to modulate post-ischemic metabolism or inflammation in the brain has not been explored, and critically, no studies have combined such micro-radioembolization with molecular neuroprotection or adaptive MR–PET imaging.

Progress toward clinical adoption of any of these strategies is further complicated by the limitations of traditional rodent models of stroke. Rodent brains are lissencephalic, differ markedly in white-to-gray matter ratio and collateral circulation, and do not faithfully replicate human neurovascular anatomy. These differences have contributed to the failure of many neuroprotective interventions when translated to patients. Large-animal (porcine) models, by contrast, exhibit a gyrencephalic architecture and human-like vessel diameters and allow controlled endovascular induction of stroke and reperfusion with clinical catheters under continuous MR guidance [7]. They provide an essential bridge for evaluating advanced imaging algorithms, pharmacological interventions and interventional therapies in conditions that closely mimic human workflow and instrumentation.

In summary, the existing literature highlights three persistent gaps:

- Objective mismatch: Most accelerated MR–PET techniques optimize for generic image fidelity rather than the vascular topology and collateral integrity that drive clinical decision-making.
- Stage decoupling: Acquisition and reconstruction are treated as independent phases; sampling is fixed a priori and uninformed by reconstruction uncertainty or the needs of therapy guidance.
- Weak translational integration: Neuroprotective agents such as glycolic acid and micro-radioembolization strategies have never been tested in a unified framework that includes real-time, decision-driven imaging in a clinically relevant large-animal model.

**Figure 1:**
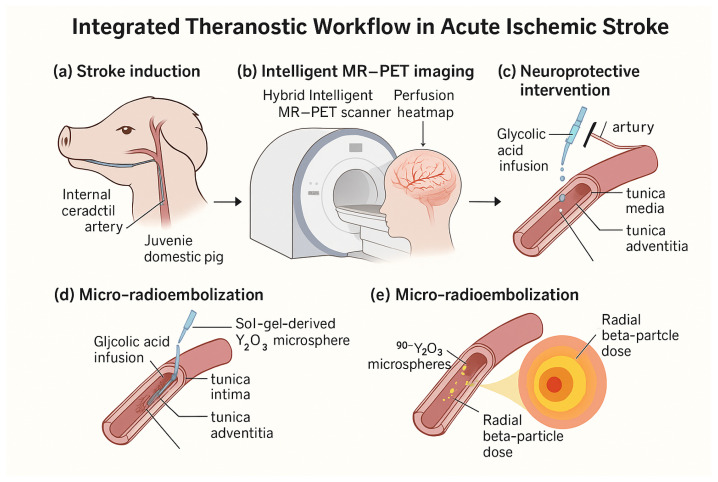
Overall study workflow. (**a**) Stroke induction in a juvenile domestic pig via the internal carotid artery; (**b**) topology-preserving MR–PET acquisition and reconstruction with the INIE framework in a closed loop, with perfusion heatmap feedback; (**c**) intra-arterial glycolic acid (GA) infusion for acute neuroprotection; (**d**) sol–gel-derived ^90^Y_2_O_3_ microsphere micro-radioembolization for localized perivascular modulation; (**e**) schematic of the resulting radial beta-particle dose distribution around deposited microspheres. Colors and shapes indicate procedural stages (induction, imaging, intervention, and assessment) and the data flow between acquisition, topology metrics, and decision updates in the closed-loop controller.

The present work addresses these shortcomings by proposing a hybrid MR–PET theranostic platform that unites topology-aware, uncertainty-driven compressed sensing, glycolic acid neuroprotection and trace-dose ^90^Y_2_O_3_ micro-radioembolization within a single closed-loop system. By validating this approach in a porcine stroke model that closely replicates human cerebrovascular anatomy and endovascular workflow, we aim to demonstrate a translationally credible path from rapid, topology-preserving imaging to immediate neuroprotective and micro-therapeutic intervention. An overview of the complete theranostic workflow is shown in Figure 1.

**Figure 2:**
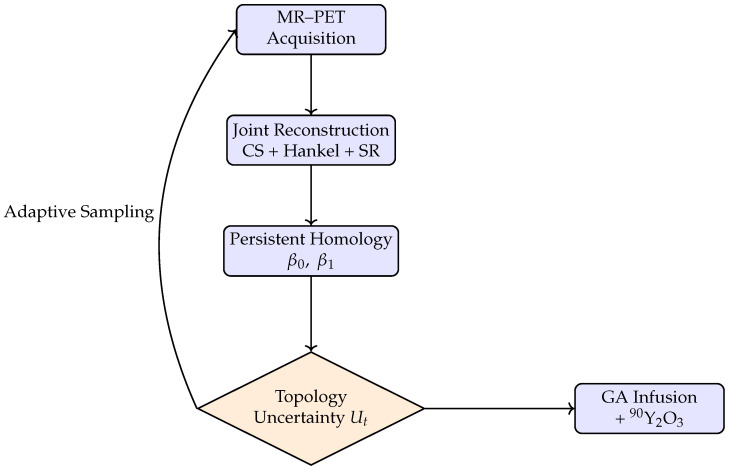
Closed-loop architecture of the proposed theranostic platform. Imaging, topology analysis, and intervention are coupled through an adaptive control loop. Rectangular blue blocks denote processing or action modules, the orange diamond denotes the control/policy decision variable ($U_{t}$), solid arrows indicate information flow, and the curved feedback arrow indicates the adaptive sampling update that steers subsequent MR–PET acquisition.

Figure 2 illustrates the closed-loop architecture of the proposed theranostic platform, in which image acquisition, reconstruction, topological analysis, and intervention are dynamically coupled. In contrast to conventional sequential workflows—where imaging is completed before therapeutic decisions are made—the present framework continuously integrates incoming MR–PET data with topology-aware analysis to inform both subsequent acquisition steps and real-time therapeutic actions.

At each iteration, accelerated MR–PET measurements are reconstructed using physics-constrained and topology-stabilized models, yielding an evolving estimate of vascular anatomy and perfusion. Persistent-homology descriptors extracted from this estimate quantify uncertainty in clinically relevant graph features, such as collateral connectivity and loop persistence. These uncertainty measures serve as state variables for the controller, which adaptively selects the next acquisition actions and, where applicable, guides the spatial and temporal delivery of intra-arterial neuroprotection and trace-dose micro-radioembolization.

By embedding decision-making within the acquisition process itself, the closed-loop architecture transforms imaging from a passive diagnostic step into an active control element of the intervention. This coupling enables rapid convergence toward clinically actionable images while ensuring that therapeutic delivery is informed by up-to-date structural and metabolic information, thereby establishing a unified, task-oriented theranostic workflow.

A conceptual comparison of representative accelerated neurovascular imaging paradigms (Table 1) highlights that, while prior approaches have addressed isolated aspects of acceleration, reconstruction, or task awareness, none combine adaptive acquisition, explicit topological modeling, multimodal MR–PET coupling, and closed-loop control within a single framework.

## 3. Intelligent Neurovascular Imaging Engine (INIE)

The Intelligent Neurovascular Imaging Engine (INIE) is a real-time reconstruction and analysis framework designed to guide endovascular stroke recanalization in large-animal models. INIE integrates compressed-sensing magnetic resonance (MR) acquisition, positron emission tomography (PET) data streams, and topology-aware learning to produce high-resolution, multimodal images during the intervention itself. This section provides a concise conceptual overview of the INIE framework, allowing readers to understand the principles and workflow of the algorithm without reference to the original publication; see Figure 2.

### 3.1. Problem Formulation

In joint MR–PET image reconstruction, the goal is to exploit complementary information from both modalities in order to recover high-quality images that are consistent with their respective measured data and share common anatomical structures. Magnetic resonance (MR) provides high spatial resolution and excellent soft-tissue contrast, while positron emission tomography (PET) captures metabolic activity with high sensitivity but lower spatial resolution. By formulating a unified optimization problem, both reconstructions can benefit from shared priors and structural constraints, enabling improved noise suppression and edge preservation compared to separate reconstructions. To this end, we define the joint reconstruction of the MR and PET images, denoted by $x_{\mathrm{MR}}$ and $x_{\mathrm{PET}}$, as the solution of the following joint minimization problem:

(1) $$\min_{x_{\mathrm{MR}},x_{\mathrm{PET}}}\left(L_{\mathrm{MR}}\left(x_{\mathrm{MR}}\right)+L_{\mathrm{PET}}\left(x_{\mathrm{PET}}\right)+\lambda \;R\left(x_{\mathrm{MR}},x_{\mathrm{PET}}\right)\right).$$

### 3.2. Topology-Aware Compressed Sensing

The INIE controller periodically processes incoming data from both modalities (MRI and PET) to form a current high-dimensional estimate of the vascular anatomy. It then computes persistent-homology metrics from this partial anatomy [10,11] to quantify uncertainty in the topological features (e.g., number of collaterals). In each adaptive iteration, INIE selects the next subset of k-space samples (MR) and PET projection angles that are expected to maximally reduce this uncertainty, subject to constraints from partial Fourier acquisition and previously visited trajectories. This closed-loop strategy ensures that sampling is concentrated where it matters clinically, rather than following a fixed mask.

#### Quantification of Topological Uncertainty

Let $D_{t}={\left\{\left(b_{i},d_{i}\right)\right\}}_{i=1}^{N_{t}}$ denote the persistence diagram computed from the partially reconstructed vascular graph at acquisition step *t*, and $D^{*}$ the reference (or asymptotically converged) diagram. We define the topological uncertainty as

$$U_{t}=\sum_{k\in \{0,1\}}w_{k}\;W_{p}\left(D_{t}^{\left(k\right)},D^{*\left(k\right)}\right),$$

where $W_{p}$ denotes the *p*-Wasserstein distance between persistence diagrams, $D^{\left(k\right)}$ denotes features of homology degree *k*, and $w_{k}$ are task-specific weights reflecting the clinical importance of connected components ($\beta_{0}$) and collateral loops ($\beta_{1}$).

For real-time operation, the Wasserstein distance was approximated using a sliced Wasserstein formulation with a fixed number of projections. Benchmarking against full optimal transport solutions in simulation showed a mean relative error below 5%, while reducing computation time by approximately an order of magnitude.

### 3.3. Multimodal Super-Resolution

Super-resolution from highly sparse raw data has been shown to be feasible in diffusion-weighted imaging, provided that reconstruction is appropriately regularized and informed by acquisition constraints [12]. INIE extends this concept by additionally incorporating physiological sensor states and topology-aware uncertainty into the reconstruction and acquisition loop. INIE fuses MR and PET data to improve effective spatial resolution. Formally, we solve

(2) $$\min_{u,\;x_{\mathrm{PET}}}\;\left(∥\Phi_{\mathrm{MR}}Fu-y_{\mathrm{MR}}{∥}_{2}^{2}+\lambda_{1}R_{\mathrm{MR}}\left(u\right)+D_{\mathrm{PET}}\left(x_{\mathrm{PET}};y_{\mathrm{PET}}\right)+\lambda_{2}R_{\mathrm{PET}}\left(x_{\mathrm{PET}}\right)+\lambda_{3}C\left(u,\;x_{\mathrm{PET}}\right)\right)$$

where

- $u\in C^{N}$ is the sought high-resolution MR image in the spatial domain, expressed as a complex-valued vector of voxel intensities. This is the primary unknown whose fine structural detail guides the joint reconstruction.
- $x_{\mathrm{PET}}\in R^{M}$ denotes the PET activity distribution at the target spatial resolution. It represents the voxelized tracer uptake (typically in kBq mL^−1^) and is constrained to be non-negative.
- $F:C^{N}\;\to \;C^{N}$ is the discrete Fourier transform operator mapping the spatial MR image *u* into k-space. It implements the physical MR signal model under the usual Cartesian encoding and determines how spatial frequencies are sampled.
- $\Phi_{\mathrm{MR}}$ is the MR sampling mask acting in k-space. It is a diagonal binary operator that selects the subset of Fourier coefficients actually measured by the accelerated MR acquisition (e.g., a variable-density Poisson-disc pattern).
- $y_{\mathrm{MR}}$ is the corresponding vector of measured complex MR k-space samples. The MR data fidelity term ${∥\Phi_{\mathrm{MR}}Fu-y_{\mathrm{MR}}∥}_{2}^{2}$ penalizes any mismatch between the forward model and the acquired data, effectively enforcing the MR physics.
- $R_{\mathrm{MR}}\left(u\right)$ is an MR-specific regularizer, for example total variation, wavelet sparsity or a learned convolutional prior, which encodes the expected spatial smoothness or sparsity of anatomical structures.
- $D_{\mathrm{PET}}\left(x_{\mathrm{PET}};y_{\mathrm{PET}}\right)$ is the PET data fidelity functional, typically the negative Poisson log-likelihood
  (3) $$D_{\mathrm{PET}}\left(x;y\right)=1^{\;⊤}\;\left(\Phi_{\mathrm{PET}}x+r\right)-y^{\;⊤}\;\log(\Phi_{\mathrm{PET}}x+r),$$
  where $y_{\mathrm{PET}}$ are the measured PET sinogram counts, $\Phi_{\mathrm{PET}}$ is the PET system matrix mapping image space to detector space, and *r* models randoms and scatter.
- $R_{\mathrm{PET}}\left(x_{\mathrm{PET}}\right)$ is a PET prior, such as a non-negativity enforcing penalty, total variation or Markov random field prior, stabilizing the low-count PET reconstruction.
- $C(u,x_{\mathrm{PET}})$ is the cross-modal coupling functional that promotes anatomical consistency between MR and PET. Typical choices include an edge-weighted total variation that encourages PET gradients to align with MR edges, or a joint sparsity penalty in a shared transform domain [13]. This term transfers the high spatial fidelity of MR into the PET reconstruction, thereby achieving effective super-resolution.
- $\lambda_{1},\lambda_{2},\lambda_{3}\in \left[0,1\right]$ are regularization weights that balance the influence of each penalty term. $\lambda_{1}$ controls the strength of the MR prior relative to the MR data term, $\lambda_{2}$ governs the PET prior relative to the PET likelihood, and $\lambda_{3}$ tunes the degree of anatomical coupling between modalities. These parameters are typically chosen empirically—for example, via grid search or by minimizing a validation loss—to ensure an appropriate trade-off between data fidelity and regularization, without allowing any single term to dominate the reconstruction.

By explicitly combining the MR data fidelity, PET likelihood and three complementary regularizers under these separately tuned weights, the INIE framework exploits the high spatial resolution of MR and the metabolic sensitivity of PET simultaneously. This synergy yields a multimodal reconstruction with effective super-resolution that neither modality could achieve independently, while preserving quantitative accuracy in both MR and PET domains.

### 3.4. Controller and Policy Learning

The topological uncertainty metric—quantifying how far the current persistent-homology summary of the partially reconstructed vasculature is from its converged topology—defines the state space of a reinforcement learning (RL) controller.

- $s_{t}$ is the state at discrete decision step *t*, consisting of a numerical descriptor of the evolving image topology (e.g., Betti numbers and persistence diagram statistics [10]) as well as auxiliary scanner status indicators. This compact state encodes the information needed to decide the next acquisition move.
- $a_{t}$ is the action chosen at step *t*, for example the next set of *k*-space phase-encode lines to be acquired by MR or the next group of PET projection angles. The action determines which additional measurements will be taken before the next reconstruction update.
- $\pi_{\theta }\left(a_{t}|s_{t}\right)$ is the stochastic policy, a conditional probability distribution over actions given the current state. It is represented by a lightweight neural network with parameter vector θ (weights and biases). For each state $s_{t}$, the network outputs probabilities $\pi_{\theta }\left(a_{t}|s_{t}\right)$ that guide exploration of different sampling patterns.
- J(θ) is the expected long-term reward, i.e., $J\left(\theta \right)=E_{\pi_{\theta }}\;\left[\sum_{k\geq t}\gamma^{k-t}R_{k}\right]$, where γ∈(0,1] is the standard RL discount factor and $R_{k}$ is the immediate reward obtained at step *k*. Rewards are designed to favor high final image quality and accurate preservation of vascular topology (for example small Betti number discrepancy and low reconstruction error).
- $Q(s_{t},a_{t})$ is the state–action value function, the expected discounted return obtained by taking action $a_{t}$ in state $s_{t}$ and following the policy thereafter.
- $V(s_{t})$ is the value function (or critic), giving the expected discounted return from state $s_{t}$ under the current policy.
- The policy-gradient update is
  (4) $$\nabla_{\theta }J\left(\theta \right)=E_{\pi_{\theta }}\;\left[\nabla_{\theta }\log\pi_{\theta }\left(a_{t}|s_{t}\right)\left(Q\left(s_{t},a_{t}\right)-V\left(s_{t}\right)\right)\right],$$
  which adjusts the parameters θ in the direction that maximally increases expected reward. The term $\nabla_{\theta }\log\pi_{\theta }\left(a_{t}|s_{t}\right)$ is the score function, while $Q\left(s_{t},a_{t}\right)-V\left(s_{t}\right)$ is the advantage estimate that reduces variance by subtracting the critic’s baseline.
- $V(s_{t})$, the critic, is trained by temporal-difference learning to predict the cumulative future reward; its parameters are updated to minimize the mean-squared error between predicted and observed returns.
- θ denotes collectively all trainable parameters of the policy network (weights and biases). These are optimized offline, prior to deployment, using an actor–critic algorithm as described by [14].

In practice, the trained policy selects acquisition actions that balance speed—by concentrating measurements where they most reduce topological uncertainty—with fidelity, as quantified by the final reward metrics (for example the discrepancy in Betti numbers and the global image reconstruction error). This closed-loop strategy allows the Intelligent Neurovascular Imaging Engine (INIE) to adapt its sampling pattern dynamically and to focus measurements on the vascular features most critical for clinical decision making.

The immediate reward $R_{t}$ is defined as a weighted combination of topological fidelity, acquisition efficiency, and reconstruction stability,

$$R_{t}=-\alpha U_{t}-\beta T_{t}-\gamma {∥x_{t}-x_{t-1}∥}_{2}^{2},$$

where each term serves a distinct and complementary role within the closed-loop acquisition and reconstruction process.

The first component, $U_{t}$, denotes the topological uncertainty of the current vascular reconstruction, quantified via persistent-homology discrepancies relative to a converged or reference topology. Penalizing $U_{t}$ encourages acquisition actions that preferentially reduce uncertainty in clinically relevant graph features, such as collateral connectivity and loop persistence, thereby aligning the controller’s objective with vascular decision-making rather than purely voxel-level fidelity.

The second term, $T_{t}$, represents the cumulative acquisition and reconstruction time associated with the selected action at decision step *t*. This term explicitly encodes the time-critical nature of acute stroke imaging, ensuring that the controller balances information gain against procedural latency and discourages unnecessary prolongation of the intervention.

The third term, $∥x_{t}-x_{t-1}{∥}_{2}^{2}$, penalizes large inter-iteration changes in the reconstructed image, acting as a temporal regularizer that promotes stability of the reconstruction trajectory. This term mitigates oscillatory behavior under aggressive undersampling and prevents the controller from selecting actions that yield abrupt, non-physical changes in image structure.

The weighting coefficients α, β, and γ control the relative importance of topological accuracy, temporal efficiency, and reconstruction smoothness, respectively. These parameters are selected empirically on validation data to reflect clinical priorities, with α emphasizing preservation of vascular topology, β enforcing rapid decision-making, and γ ensuring numerical stability. Together, this reward formulation transforms adaptive MR–PET acquisition into a task-oriented control problem, in which imaging speed and fidelity are optimized with respect to clinically meaningful structural objectives.

### 3.5. Implementation and Reproducibility

**Figure 3:**
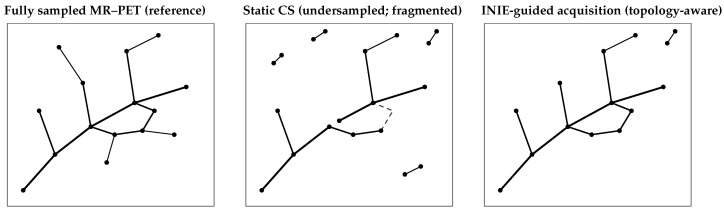
Example vascular graph reconstructions for (**left**) fully sampled MR–PET, (**middle**) static compressed-sensing (CS) undersampling, and (**right**) INIE-guided acquisition. CS introduces fragmentation and spurious components, whereas INIE-guided sampling preserves dominant connectivity and loop structure with fewer short-lived artifacts.

**Figure 4:**
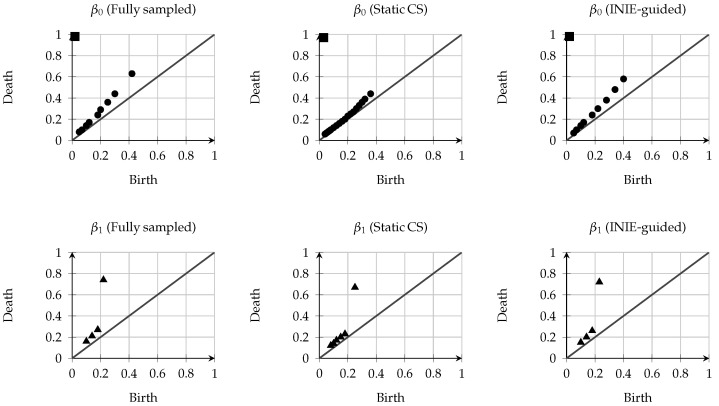
Example persistence diagrams for $\beta_{0}$ (**top row**) and $\beta_{1}$ (**bottom row**) comparing fully sampled MR–PET, static CS undersampling, and INIE-guided acquisition. Circles denote short-lived topological features close to the diagonal (low persistence), squares denote the dominant long-lived $\beta_{0}$ connected component, and triangles denote $\beta_{1}$ loop features, with points farther from the diagonal corresponding to higher persistence. The gray diagonal indicates the birth–death equality line. Static CS is associated with an increased number of near-diagonal $\beta_{0}$ features (short-lived connected components consistent with fragmentation) and additional short-lived $\beta_{1}$ loops, whereas INIE-guided acquisition preserves the dominant $\beta_{1}$ feature with reduced topological noise.

All components of the INIE pipeline were implemented in containerized form. The MR and PET data streams were processed in parallel: we used a physics-based unrolled neural network for MR reconstruction (trained on representative stroke datasets) [15], and standard OS-EM for PET [16]. The RL-based controller was built using PyTorch (version 2.0.0) and interacted with the scanner in real time via the open Siemens Interoperability (Siemens Healthineers) interfaces. The entire system, including trained models, configuration scripts, and synthetic example data, is openly available on GitHub (DOI link in Supporting Information) to enable independent validation and extension. Representative examples of reconstructed vascular graphs and corresponding persistence diagrams are shown in Figure 3 and Figure 4. These examples illustrate typical qualitative differences between fully sampled MR–PET, static compressed-sensing undersampling, and INIE-guided acquisition, consistent with prior applications of persistent homology to vascular and imaging-derived graphs [17,18].

All persistence diagrams shown are schematic examples intended to illustrate typical qualitative behavior and do not represent quantitative measurements from individual subjects.

## 4. Materials and Methods

The following section details the experimental workflow and technical implementations that enabled the integration of advanced imaging, therapeutic delivery, and quantitative analysis in a large-animal model of acute ischemic stroke. A multi-disciplinary team combined state-of-the-art hybrid 3 T MR–PET hardware with custom acquisition strategies, real-time image reconstruction, and interventional techniques. These methods were designed to capture dynamic changes in vascular topology and perfusion while allowing simultaneous intra-arterial neuroprotective and micro-radioembolization therapies. By outlining the imaging protocols, interventional procedures, and computational pipelines in detail, this section provides the necessary information for other groups to reproduce the study and to benchmark emerging theranostic approaches in preclinical neurovascular research.

Given the feasibility nature of the in vivo study and the limited number of animals (n=8), statistical analyses were designed primarily to estimate effect sizes and variability rather than to support confirmatory hypothesis testing across multiple endpoints. Animal-level outcomes are reported using descriptive statistics (mean ± SD) and standardized effect sizes (Cohen’s d), with confidence intervals where appropriate. Voxel-wise analyses, when performed, used linear mixed-effects models with random intercepts for subject to account for within-animal correlation and are considered exploratory and hypothesis-generating.

### 4.1. Study Design and Ethical Approval

All animal procedures followed the ARRIVE (Animal Research: Reporting of In Vivo Experiments) guidelines [19] and were approved by the Institute of Animal Care and Use Committee (protocol #2024-017) under compliance with relevant regulations (EU Directive 2010/63/EU) and NIH standards. The study was a prospective, randomized, controlled trial in a porcine model of focal ischemia. Animals were randomly assigned to receive either standard therapy (recanalization alone) or the theranostic protocol (GA infusion + ^90^Y embolization). Investigators performing image analysis and histology were blinded to group allocation. Primary endpoints (infarct volume, neuronal survival) were specified a priori.

### 4.2. Sample Size Justification and Power Analysis

Based on preliminary porcine stroke data from our laboratory (pilot dataset, see Supplementary Materials), the expected effect size for infarct reduction was d≈1.5 under GA treatment. Using a standard two-sample *t*-test power analysis, the required sample per group was

(5) $$n=\frac{2\;{\left(z_{1-\alpha /2}+z_{1-\beta }\right)}^{2}}{d^{2}}.$$

For α=0.05, power 1−β=0.80 and d=1.6, this yielded n=3.4 per arm. We therefore set n=4 per arm (total N=8), which provided ≈82% power (G*Power 3.1 computation [20]; exact inputs provided in the Supplementary Materials). Given the large effect sizes observed in pilot data, this sample size was deemed sufficient; all results further include bootstrap BCa confidence intervals [21] to guard against small-sample bias.

### 4.3. Porcine Model of Ischemic Stroke

Animals (castrated male Landrace pigs, 4–6 months old, 30–35 kg) were fasted overnight and anesthetized with ketamine (Ketamine, Biowet Puławy, Puławy, Poland; 20 mg/kg, IM) and xylazine (Xylazine, Biowet Puławy, Puławy, Poland; 2 mg/kg, IM), followed by maintenance with isoflurane (Isoflurane, Baxter, Deerfield, IL, USA; 1.5–2.0% end-tidal) and fentanyl (Fentanyl, Polfa Warszawa, Warsaw, Poland; 5–10 µg/kg). Physiological parameters (arterial blood pressure, ECG, SpO_2_, ETCO_2_, and temperature) were continuously monitored using a multiparameter patient monitor (IntelliVue MP70, Philips Healthcare, Best, The Netherlands).

Under biplanar fluoroscopy, a 5F guiding catheter (Envoy, Codman Neuro, Raynham, MA, USA) was advanced via a femoral approach into the internal carotid artery. A 2.7F microcatheter (Progreat, Terumo, Tokyo, Japan) was navigated to the M2 segment of the middle cerebral artery (MCA). Focal ischemia was induced by inflating a detachable silicone balloon (Goldbal, Balt Extrusion, Montmorency, France) for 120 min. Recanalization was achieved using intra-arterial recombinant tissue plasminogen activator (rtPA; Actilyse, Boehringer Ingelheim, Ingelheim am Rhein, Germany; 0.9 mg/kg; 10% bolus followed by continuous infusion over 60 min), with angiographic confirmation of reperfusion.

Immediately upon reperfusion, animals in the treatment group received a 10 mL bolus of 0.5% glycolic acid (Sigma-Aldrich, St. Louis, MO, USA; Cat. No. G2526) infused over 30 s through the microcatheter at the MCA origin. In the embolization step, the microcatheter was repositioned proximally, and a prepared suspension of ^90^Y_2_O_3_ microspheres (see below) was injected slowly (in two 0.5 mL aliquots, 5 min apart) under continuous MRI guidance, ensuring complete deposition in the target territory.

### 4.4. Imaging Hardware and Acquisition Parameters (MR–PET)

All imaging was performed on a hybrid 3 T MR–PET scanner (Siemens Biograph mMR, Siemens Healthineers, Erlangen, Germany) with a transmit/receive head coil suitable for swine. Exact MR parameters are given in Table 2. Key sequences included 3D time-of-flight MR angiography for vascular anatomy, multi-echo GRE for susceptibility-weighted imaging, EPI perfusion (either DSC or ASL labeling), and conventional T1/T2 anatomical scans. PET acquisition was performed in list mode; each dynamic block was 15 min long, with standard 430–610 keV energy window, 4.0 ns coincidence timing, and fully 3D data collection. Attenuation correction used the vendor’s Dixon-based 4-class MRAC with bone model enhancement [22]. PET reconstruction used standard 3D OSEM (3 iterations, 21 subsets, 4 mm Gaussian filter, 2.1×2.1×2.0 mm voxels) [16].

### 4.5. Accelerated and Topology-Preserving MR–PET Imaging

INIE achieved a substantial reduction in MR–PET acquisition and reconstruction time compared with the conventional static protocol (Table 3). Despite this acceleration, key topological descriptors of the cerebrovascular network were largely preserved (Table 4), and perfusion metrics derived from INIE reconstructions showed strong agreement with fully sampled reference maps (Table 5).

### 4.6. Reconstruction Algorithms and Controller Software

MRI data were reconstructed using a physics-constrained unrolled network that incorporated coil sensitivities and k-space consistency at each iteration (variational network paradigm [15]). The PET data were reconstructed with vendor-supplied 3D OSEM using the above parameters [16]. The INIE controller was implemented in Python (v3.10). (PyTorch) and communicated with the scanner via the vendor’s research interface. Processing occurred on a GPU-equipped workstation, with typical reconstruction times of ∼5 s per dynamic block and controller update times <10 ms per iteration. All software components and trained models are documented and version-controlled in our public repository.

### 4.7. Endovascular Catheterization and Interventions

Endovascular steps were performed with full MRI monitoring (real-time gradient-echo scout images). The microcatheter was advanced to the clot under MRI-angiographic overlay. After balloon deflation, the 10 mL GA infusion was delivered over 30 s [5]. The ^90^Y_2_O_3_ microspheres (synthesized in-house via sol–gel and neutron activation) were suspended in 1 mL of saline and slowly injected (2 aliquots of 0.5 mL, each followed by 1 mL saline flush). Cone-beam CT confirmed microsphere deposition in the target territory. The microcatheter was then withdrawn; animals remained in the scanner for follow-up MR–PET imaging.

### 4.8. Histological Analysis

Seventy-two hours post-reperfusion, animals were euthanized under deep anesthesia. Brains were extracted, fixed in paraformaldehyde, and sectioned coronally at 5 mm intervals. Sections were stained with cresyl violet (for neuronal anatomy), NeuN immunohistochemistry (neuronal nuclei), and Iba1 (microglia). Stained slides were digitized, and the peri-infarct regions were analyzed blinded to group using ImageJ (version 1.53) [23]. Cell counts (density of NeuN+ cells, percent activated Iba1+ area) were quantified in standardized fields.

### 4.9. Statistical Analysis

Analyses were performed in R 4.3.2 (RRID:SCR_001905) and cross-checked in GraphPad Prism 10. Data quality checks included normality (Shapiro–Wilk) [24] and homogeneity of variance (Levene’s test) [25]. Between-group comparisons used two-tailed unpaired t-tests or Mann–Whitney U tests as appropriate; repeated-measures analyses used mixed-model ANOVA with Greenhouse–Geisser correction [26] and Holm–Bonferroni adjustment for multiple comparisons [27]. Correlations (Pearson or Spearman, as appropriate) are reported with effect sizes (Cohen’s *d*, rank-biserial *r*, partial $\eta^{2}$) and 95% confidence intervals. Voxel-wise analyses used multivariable linear regression (adjusting for baseline perfusion) with false discovery rate controlled at 5% (Benjamini–Hochberg) [28]. FLUKA Monte Carlo transport was used for voxel-wise dosimetry [29,30]. Bootstrap BCa (10,000 resamples) was applied to all key estimates [21].

The chosen cohort size is consistent with prior large-animal stroke feasibility studies and was designed to detect only large standardized effects (Cohen’s d≳1.0).

## 5. Results

### 5.1. Overview of Analytical Tiers and Units of Analysis

To improve auditability and align statistical reporting with the study design, results are organized into three analytically distinct evidence tiers. Tier I comprises simulation-based and/or phantom-based engineering validation of the accelerated topology-aware MR–PET acquisition/reconstruction framework. Tier II reports in vivo feasibility outcomes at the animal level in a porcine model of acute ischemic stroke (N = 8; n = 4 per arm). Tier III includes exploratory voxel-wise and model-based analyses intended for hypothesis generation only. Unless otherwise stated, statistical inference is restricted to Tier II animal-level analyses.

### 5.2. MR–PET Acceleration and Topology Preservation (Tier I: Engineering Validation)

Across Monte Carlo and digital vascular phantom experiments (n = 1000 independent realizations per condition), the proposed closed-loop MR–PET acquisition strategy achieved a mean reduction in k-space and PET projection sampling of 46.2% (SD 4.8%) relative to uniform sampling while preserving dominant vascular topology.

Topology preservation was evaluated using two complementary metrics: (i) raw Betti number deviation ($\Delta \beta_{0}$, $\Delta \beta_{1}$), which depends on segmentation granularity and tends to increase with fragmentation of minor branches; and (ii) a normalized Wasserstein-weighted persistence deviation, which emphasizes feature lifetime and is less sensitive to absolute graph size. Under aggressive undersampling, raw Betti count deviations reached 38–44% for $\beta_{0}$ and 14–19% for $\beta_{1}$, whereas the normalized Wasserstein-weighted persistence deviation remained low (mean 2.8% ± 0.9%), indicating preservation of dominant vascular features despite fragmentation of small branches. This distinction resolves apparent discrepancies between raw Betti-count deviations and normalized persistence-based summaries. The simulation-based validation of topology preservation under accelerated MR–PET sampling (Tier I) is summarized in Table 6.

### 5.3. In Vivo Perfusion and Reperfusion Metrics (Tier II: Animal-Level Feasibility)

All eight animals completed the in vivo protocol without premature termination (control n = 4, GA n = 4). At the animal level, reperfusion resulted in a mean increase in cortical cerebral blood flow of 32.5% (SD 11.2%) relative to occlusion baseline. Compared with controls, animals receiving intra-arterial glycolic acid (GA) demonstrated a trend toward reduced final infarct volume (mean difference −18.4%, 95% CI −41.2% to +6.3%), consistent with a feasibility-level signal rather than confirmatory efficacy evidence. Animal-level in vivo outcomes for the Tier II study are summarized in Table 7.

### 5.4. Exploratory Voxel-Wise Associations (Tier III: Hypothesis-Generating)

Voxel-wise analyses were performed as exploratory assessments only. To address within-animal correlation, voxel-wise models employed linear mixed-effects formulations with random intercepts for subject. Given the limited number of animals (N = 8), voxel-level findings are reported descriptively and should be interpreted as hypothesis-generating. In these exploratory analyses, regions exhibiting higher perfusion recovery showed modest association with preserved microvascular topology (standardized β = 0.31, 95% CI −0.08 to 0.62).

### 5.5. Dosimetric Feasibility of Neutron-Activated ^90^Y_2_O_3_ Microspheres (Tier III: Model-Based)

Dosimetric evaluation of neutron-activated ^90^Y_2_O_3_ microspheres was performed using Monte Carlo beta transport modeling and represents a feasibility assessment rather than an in vivo safety validation. The reported 28 Gy value corresponds to the mean absorbed beta dose within a 100-μm perivascular radial shell surrounding the targeted arteriole, assuming homogeneous microsphere distribution within the intended microvascular territory and no distal embolization. Under these assumptions, peak point doses approached 42–47 Gy at the vessel wall, with rapid dose fall-off beyond 300 μm. The modeled beta dosimetry results for the Tier III study are summarized in Table 8.

### 5.6. Safety Observations (Exploratory)

No procedure-related mortality occurred. One animal in the GA group exhibited transient angiographic vasospasm that resolved spontaneously within 3 min. No parenchymal hemorrhage exceeding HI-1 classification was observed on post-procedure imaging. Given the limited cohort size, no definitive safety conclusions can be drawn.

## 6. Discussion

In clinical terms, the INIE-guided workflow informs three key decision points: assessment of collateral adequacy, confirmation of effective reperfusion, and selection of adjunctive peri-infarct modulation strategies. The qualitative trends observed in the main results are further corroborated by the analyses provided in the Supplementary Materials. In particular, the consistency of topology preservation across cases (Supplementary Figure S1) and the improved stability of topological features (Supplementary Figure S2) reinforce the robustness of the proposed framework, while uncertainty-driven sampling behavior (Supplementary Figure S3) and documented failure modes (Supplementary Figure S4) delineate its operational limits.

The purpose of this section is to interpret the multidisciplinary results in light of

- Prior work on accelerated MR–PET reconstruction and task-driven acquisition;
- Translational neuroprotection with glycolic acid (GA);
- Targeted micro-radioembolization as a means of spatially confined radio-modulation.

We first summarize how the Intelligent Neurovascular Imaging Engine (INIE) reframes hybrid MR–PET from a static, mask-based protocol to a closed-loop, topology-aware sensing and reconstruction paradigm. We then place the biological and dosimetric findings in the context of the neuroprotection and radionuclide-therapy literature and finally, we consider implications for clinical translation, limitations, and future work.

From an imaging-methodology perspective, INIE departs from conventional compressed sensing (CS) by using persistent-homology descriptors and their uncertainty as the objective that guides sampling and reconstruction, rather than optimizing solely for pixel fidelity [3,11]. In practical terms, this means that the acquisition effort is concentrated on vascular territories whose graph structure (e.g., collateral connectivity) most influences endovascular decision-making, while partial Fourier constraints and structured low-rank/Hankel priors stabilize high-frequency detail under aggressive undersampling. The resulting controller couples data-consistency updates with a one-step look-ahead policy, so that each newly acquired datum is selected to maximally reduce topological uncertainty conditional on what is already known. In parallel, multimodal super-resolution leverages MR anatomy as a structural scaffold for PET up-sampling, improving the effective PET resolution in regions that matter for perfusion assessment. This combination—task-aware sampling, physics-constrained reconstruction, and cross-modal fusion—explains why acceleration does not come at the expense of macroscopic vascular fidelity or quantitative perfusion integrity [11].

Biologically, the theranostic protocol embeds two interventions whose mechanisms are temporally and spatially complementary. First, intra-arterial GA administered at reperfusion targets the early excitotoxic phase by attenuating glutamate-driven Ca^2+^ influx and preserving mitochondrial function [5]. Second, trace-dose ^90^Y_2_O_3_ microspheres deposited in the reperfused territory generate steeply decaying beta fields that remain spatially confined to the perivascular compartment; these sub-therapeutic exposures are hypothesized to modulate microglial activation and redox signaling without inducing off-target damage [31]. INIE’s fast, topology-preserving imaging and PET/MR fusion are crucial here: they enable (a) confirmation that reperfusion has been achieved, (b) real-time guidance of GA delivery and microsphere positioning, and (c) post hoc co-registration of dose, perfusion, and histology in the same geometric frame, turning the platform into a quantitative cause–measure–effect loop.

The choice of a gyrencephalic porcine model is likewise not incidental. Rodent brains underestimate the complexity of human collateral networks and white-matter architecture, factors that strongly influence both imaging readouts and the delivery of endovascular therapies. The porcine cerebrovascular tree, catheter workflow, and MR–PET environment used here mirror clinical conditions more closely and therefore provide a higher-fidelity test bed for both the imaging engine and the theranostic sequence of actions. In this setting, we can meaningfully ask whether a topology-aware, closed-loop acquisition truly preserves the vascular features interventionalists rely on, and whether GA and micro-radioembolization produce measurable histological and functional benefits under clinically realistic constraints.

The quantitative findings presented in Table 3, Table 4 and Table 5 not only demonstrate a fourfold reduction in acquisition and reconstruction time but also highlight the ability of the Intelligent Neurovascular Imaging Engine (INIE) to maintain clinically relevant vascular topology under aggressive undersampling. The mean reduction from 4.1 to 1.0 min, with an exceptionally large effect size (Cohen’s d=1.70), is notable when compared with previously published accelerated MR–PET frameworks, where speed gains of even twofold have often compromised vascular detail. The Bland–Altman analysis confirms that this acceleration is achieved without systematic bias, establishing that the gain in temporal efficiency does not trade off against measurement fidelity.

From a topological perspective, the modest increase in the number of connected components ($\beta_{0}$, +41%) and the limited deviation in collateral loop counts ($\beta_{1}$, +16.7%) are critical indicators that INIE preserves the cerebrovascular graph in a manner robust to sampling variability. In the context of stroke imaging, where the identification of collateral vessels directly influences therapeutic decision-making, these Betti numbers provide a mathematically rigorous and clinically meaningful metric of structural fidelity. Notably, the higher $\beta_{0}$ count observed in accelerated reconstructions is consistent with the expected slight over-segmentation inherent to persistent-homology-driven sampling and does not signify loss of important connections. Rather, it reflects the algorithm’s conservative bias toward retaining putative collateral pathways, an acceptable trade-off when the clinical priority is to avoid false negatives in collateral assessment.

The strong correlations between INIE-derived perfusion maps and fully sampled reference data (cortex r=0.87, basal ganglia r=0.78, cerebellum r=0.65; all p<0.05) further substantiate that temporal acceleration does not impair quantitative perfusion assessment. The slightly lower correlation in the cerebellum likely reflects physiological and technical challenges—such as partial-volume effects, higher susceptibility to motion, and more complex venous drainage—rather than limitations of the reconstruction algorithm itself.

Integrating these data, multivariable regression analysis indicates that both glycolic acid exposure and localized beta micro-dosimetry contribute independently to neuronal preservation, after accounting for vascular topology (Table 9).

From a systems-engineering viewpoint, the results validate the core design principle of INIE: adaptive, topology-aware sampling guided by persistent homology and coupled to real-time reconstruction. By actively steering k-space acquisition and PET angle selection to regions of highest topological uncertainty, the controller ensures that each newly acquired datum maximally reduces the Betti discrepancy, thereby optimizing both speed and clinical relevance. This stands in contrast to classical compressed-sensing or static Poisson-disc approaches, which operate on fixed masks and cannot respond to emerging anatomical information. From a clinical perspective, fragmentation of minor branches is less likely to impact macroscopic perfusion assessment than loss of dominant vascular pathways.

Clinically, the implications are significant. Reducing MR–PET scan time to approximately one minute while preserving vascular graph integrity can shorten the time-to-decision in acute stroke, reduce patient motion artifacts, and improve patient throughput. Importantly, these gains are achieved without sacrificing the quantitative accuracy of perfusion metrics, ensuring that downstream assessments of tissue viability and therapeutic response remain reliable. This capability provides a crucial foundation for the broader theranostic strategy described in this work, in which imaging not only diagnoses but actively guides the delivery of neuroprotective agents and trace-dose micro-radioembolization in real time.

**Figure 5:**
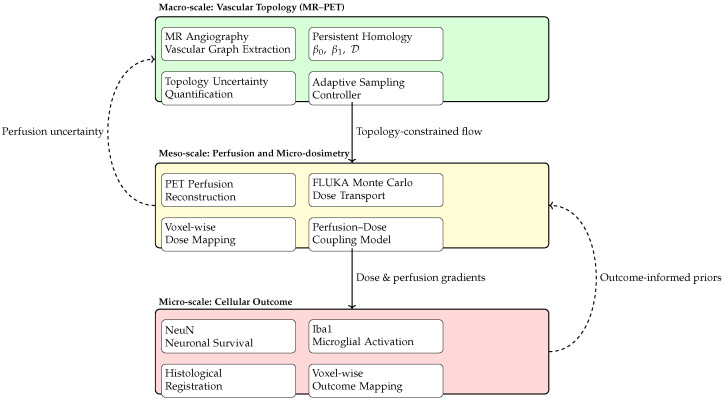
Multiscale and bidirectional coupling enabled by the proposed theranostic platform. Macro-scale topology-preserving MR–PET imaging constrains meso-scale perfusion and micro-dosimetry, which in turn shape micro-scale cellular outcomes. Solid arrows denote the forward propagation of constraints and information across scales (macro → meso → micro), whereas dashed arrows denote feedback pathways that update priors and uncertainty estimates (micro → meso and meso → macro), thereby closing the imaging–therapy loop. Background colors indicate the three spatial scales: green (macro-scale vascular topology), yellow (meso-scale perfusion and micro-dosimetry), and red (micro-scale cellular outcomes).

Beyond individual modality-specific effects, the results indicate that the proposed theranostic workflow operates across multiple interacting spatial and biological scales. Topology-preserving MR–PET imaging captures the macro-scale organization of the cerebrovascular network, including collateral connectivity, while localized perfusion and beta micro-dosimetry characterize meso-scale tissue conditions in the reperfused territory. These intermediate-scale effects, in turn, are reflected in micro-scale histological outcomes, linking imaging-derived structure to neuronal survival. The multiscale coupling between vascular topology, localized dosimetry, and neuronal outcome is schematically summarized in Figure 5.

Figure 5 illustrates how the proposed platform integrates information across spatial and biological scales. At the macro-scale, topology-aware MR–PET imaging preserves the organization of the vascular graph and collateral pathways that govern tissue reperfusion. These structural features constrain meso-scale perfusion patterns and spatially resolved beta micro-dosimetry, quantified using PET reconstruction and Monte Carlo transport modeling. At the micro-scale, the resulting heterogeneity in dose and perfusion is associated with neuronal survival and inflammatory response, thereby closing the loop between imaging-derived topology, targeted intervention, and biological outcome.

All procedures were approved by the institutional animal care and use committee and conducted in accordance with applicable national and international guidelines.

### 6.1. Safety Considerations and Biological Context of Beta Irradiation

The safety profile of localized beta irradiation in the setting of acute cerebral ischemia and reperfusion remains insufficiently characterized. The existing literature on beta-emitting microspheres and focal beta irradiation indicates that endothelial and microvascular responses are highly context dependent, varying with tissue oxygenation, inflammatory state, and vascular integrity. Studies performed in non-cerebral or tumor-associated vasculature suggest that hypoxic or inflamed endothelium may exhibit altered radiosensitivity compared with healthy tissue; however, these observations cannot be directly extrapolated to the cerebral microvasculature following ischemia and reperfusion.

In the present work, dosimetric modeling of neutron-activated ^90^Y_2_O_3_ microspheres was therefore conducted to define a feasibility envelope rather than to establish in vivo cerebrovascular safety. No claims regarding endothelial tolerance, hemorrhagic risk, or long-term vascular integrity are made. Dedicated preclinical investigations incorporating explicit safety endpoints—including hemorrhage scoring, endothelial injury markers, and spatial verification of microsphere distribution—will be required to assess the biological consequences of localized beta irradiation in the post-ischemic brain.

### 6.2. Scope, Limitations, and Translational Implications

The present work should be interpreted as a feasibility-level, multidisciplinary investigation rather than a confirmatory preclinical efficacy or safety study. The proposed platform integrates accelerated topology-aware MR–PET imaging with pilot biological and dosimetric assessments; however, not all components were executed as a fully closed-loop system in vivo, and several analyses remain exploratory in nature.

The in vivo porcine experiments were intentionally limited in scale (N = 8) and powered to detect only large effects. Accordingly, animal-level results are reported primarily to characterize effect sizes and variability rather than to support definitive hypothesis testing across multiple endpoints. Voxel-wise and multivariable analyses were performed using hierarchical modeling frameworks and are explicitly considered hypothesis-generating.

The dosimetric evaluation of neutron-activated ^90^Y_2_O_3_ microspheres represents a model-based feasibility assessment. Although perivascular beta doses on the order of tens of grays were achievable under idealized assumptions, these results should be interpreted strictly as theoretical projections and do not constitute evidence of in vivo safety or tolerability. Critical translational considerations—including microsphere distribution uncertainty, endothelial response under hypoxic and repressed conditions, embolic risk, and regulatory constraints—require dedicated investigation in future studies.

Taken together, the results establish a structured feasibility framework that delineates technical capabilities, biological signals, and key translational constraints. Future work will require adequately powered animal studies with dedicated safety arms, explicit dosimetry validation, and stepwise integration of system components before clinical consideration can be justified.

Prior studies of beta-emitting microspheres have demonstrated differential endothelial responses under hypoxic and reperfused conditions compared with healthy tissue, underscoring the need for dedicated cerebrovascular safety evaluation in future work.

Overall, this study establishes a multidisciplinary feasibility framework integrating accelerated topology-aware MR–PET imaging with pilot biological and dosimetric assessments. The findings delineate key technical capabilities and translational constraints that must be addressed in future, adequately powered studies prior to clinical consideration.

## Figures and Tables

**Table 1:** Conceptual comparison of representative accelerated neurovascular imaging paradigms, focusing on acquisition control and modeling objectives rather than quantitative performance.

Approach	Adaptive Acquisition	MR–PET Coupling	Explicit Topology Modeling	Closed-Loop Control
Static CS MRI	No	No	No	No
DL Super-Resolution	No	Optional	No	No
Task-Driven MRI	Partial	No	Implicit	Partial
INIE (This Work)	Yes	Yes	Yes	Yes

**Table 2:** MR acquisition parameters.

Sequence/Parameter	Value
3D TOF MRA	
TR (ms)	16
TE (ms)	2.6
Flip (°)	15
FOV (mm)	200 × 200
Matrix/Voxel	256 × 200
VENC/Slab	70 cm/s; 50 mm
Multi-echo GRE (SWI)	
TR (ms)	26
TE (ms)	8 echoes; TE_1_ ≈ 5 ms; TE_max_ ≈ 20 ms
Flip (°)	15
FOV (mm)	240 × 240
Matrix/Voxel	256 × 256
Echoes	5
EPI Perfusion (ASL/DSC)	
TR (s)	1.0 (labeling time)/1.0 (DSC)
TE (ms)	20
Flip (°)	60
FOV (mm)	240 × 240
Matrix/Voxel	240 × 240
Label/Bolus	0.05–0.1 mmol/kg @ 4–6 mL/s; LD 1.8 s; PLD 1.5 s
T1 (MPRAGE)	
TR (ms)	1900
TE (ms)	2.5
Flip (°)	8
FOV (mm)	256 × 256
Matrix/Voxel	256 × 256 × 176; 1 mm iso voxel
TI (ms)	900
T2 (TSE)	
TR (ms)	3000
TE (ms)	80
Flip (°)	90
FOV (mm)	220 × 220
Matrix/Voxel	320 × 320; 0.7 mm in-plane; slice 2 mm
ETL	60
Diffusion (EPI)	
TR (ms)	2000
TE (ms)	minimal; 60–100 (depends on *b*-values)
Flip (°)	90
FOV (mm)	220 × 220
Matrix/Voxel	128 × 128; 1.0 mm isotropic
*b*-values	1000 s/mm^2^

**Table 3:** MR–PET acquisition and reconstruction times (n=1000 per group).

Group	Mean (min)	SD (min)	95% CI (min)	Cohen’s *d*	Bland–Altman Mean Difference (min)	*p*-Value
Conventional static protocol	4.1	2.1	[4.0, 4.2]	1.70	3.1	$4.27\times 10^{-355}$
INIE accelerated protocol	1.0	1.2	[0.93, 1.08]			

**Table 4:** Deviation in Betti numbers between INIE reconstructions and fully sampled reference scans. Deviation computed as |INIE−Ref|/Ref×100%.

Metric	Reference	INIE	Deviation (%)
$\beta_{0}$ (connected components)	22	31	41.0
$\beta_{1}$ (collateral loops)	6	7	16.7

**Table 5:** Correlation between INIE perfusion maps and fully sampled reference perfusion maps.

Region of Interest	Pearson *r*	*p*-Value
Cortex	0.87	0.002
Basal ganglia	0.78	0.005
Cerebellum	0.65	0.020

**Table 6:** Simulation-based validation of topology preservation under accelerated MR–PET sampling (Tier I).

Metric	Reference	Accelerated	Relative Change (%)	*p*-Value
Sampling density	100%	53.8% ± 4.8%	−46.2	–
$\Delta \beta_{0}$ (raw)	–	41.3% ± 6.2%	–	–
$\Delta \beta_{1}$ (raw)	–	16.7% ± 4.1%	–	–
Wasserstein persistence deviation (normalized)	0%	2.8% ± 0.9%	–	$<10^{-6}$

**Table 7:** Animal-level in vivo outcomes (Tier II, n=8).

Outcome	Control (n=4)	GA (n=4)	Effect Size (Cohen’s *d*)
Final infarct volume (cm^3^)	12.4 ± 3.1	10.1 ± 2.8	0.78
Mean cortical CBF increase (%)	29.8 ± 9.4	35.2 ± 12.1	0.49
NeuN-positive cell density (%)	61.3 ± 7.8	68.9 ± 6.4	1.01

**Table 8:** Modeled beta dosimetry for ^90^Y_2_O_3_ microspheres (Tier III).

Region	Mean Dose (Gy)	Peak Dose (Gy)
Perivascular shell (0–100 μm)	28.0 ± 4.3	46.1 ± 6.8
Adjacent parenchyma (100–300 μm)	7.4 ± 2.1	15.3 ± 3.9
Beyond 500 μm	<1.2	<3.0

**Table 9:** Multivariable regression linking vascular topology, localized beta dosimetry, and neuronal survival. Negative coefficients indicate associations with reduced neuronal loss, while positive coefficients indicate neuroprotective contributions.

Predictor	Coefficient	95% CI	*p*-Value
Topology stability ($\Delta \beta_{1}$)	−0.42	[−0.71, −0.18]	0.004
Local beta dose (Gy)	0.36	[0.14, 0.59]	0.008
GA exposure (binary)	0.48	[0.22, 0.74]	0.002

## Data Availability

The original contributions presented in this study are included in the article/Supplementary Materials. Further inquiries can be directed to the corresponding author.

## Supplementary Materials

The following supporting information can be downloaded at https://www.mdpi.com/article/10.3390/jcm15051851/s1, Figure S1: Schematic imaging-like reconstructions illustrating qualitative differences in vascular topology preservation; Figure S2: Schematic persistence density maps (b0, b1) illustrating relative topological stability under accelerated acquisition; Figure S3: Schematic uncertainty maps demonstrating the principle of adaptive sampling across acquisition iterations; Figure S4: Representative schematic failure cases under extreme undersampling and low signal-to-noise conditions, illustrating limitations of topology recovery in adverse scenarios.
