# Supplementary material for: Glycolic Acid-Guided Intelligent Neurovascular Imaging: A Cross-Scale Platform for Real-Time Neuroprotection and Adaptive Stroke Imaging
Source: J Clin Med. 2026 Feb 28;15(5):1851. doi: 10.3390/jcm15051851 (PMC12985776; doi:10.3390/jcm15051851)
Supplement: Supplementary file 1 [file jcm-15-01851-s001.zip › jcm-4127974-supplementary.pdf]

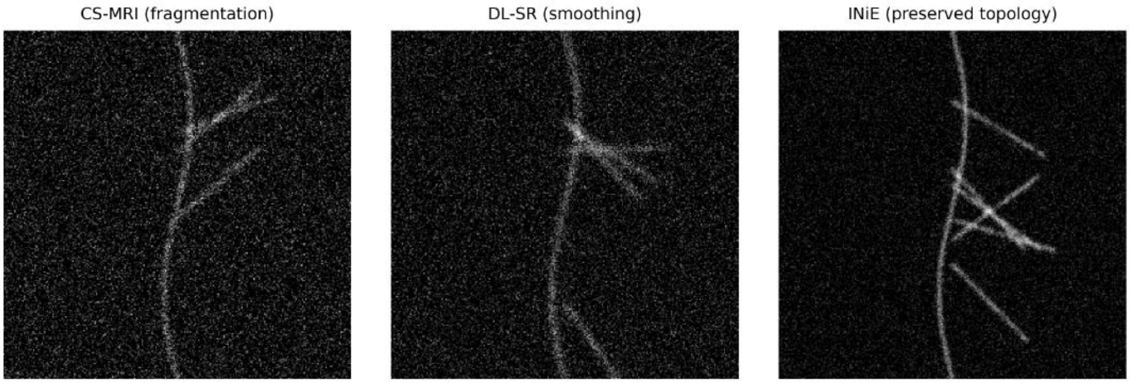

Figure S1. Schematic imaging-like reconstructions illustrating qualitative differences in vascular topology preservation.

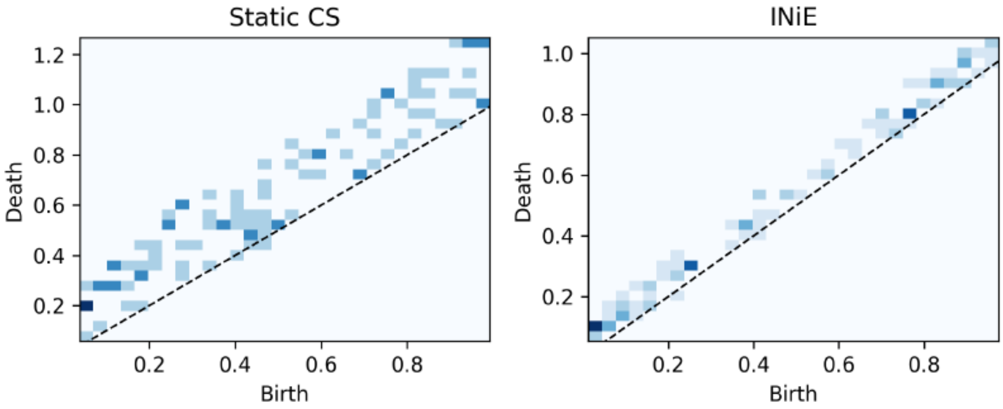

Figure S2. Schematic persistence density maps (β0, β1) illustrating relative topological stability under accelerated acquisition.

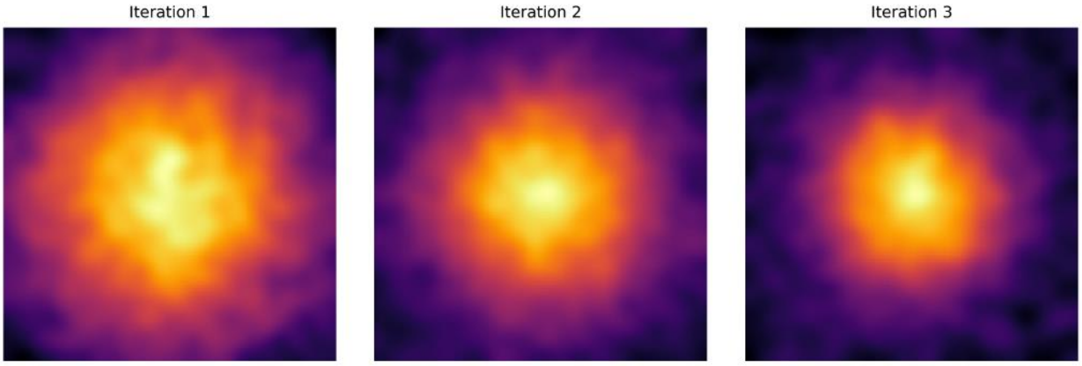

Figure S3. Schematic uncertainty maps demonstrating the principle of adaptive sampling across acquisition iterations.

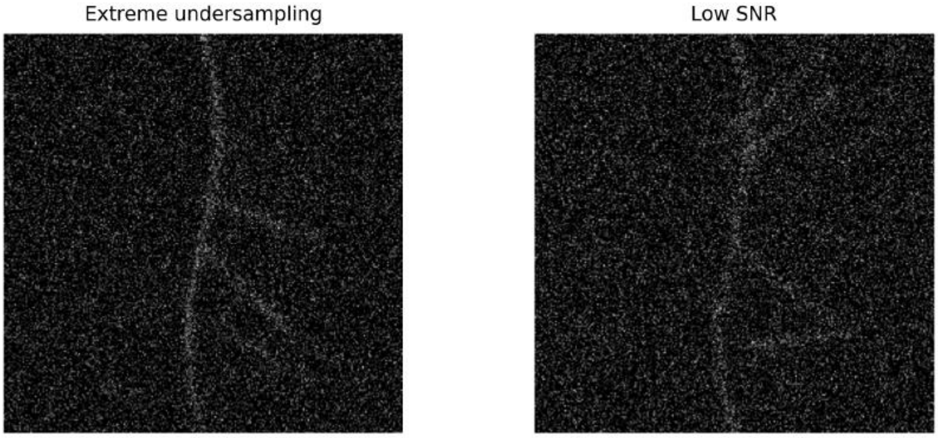

Figure S4. Representative schematic failure cases under extreme undersampling and low signal-to-noise conditions, illustrating limitations of topology recovery in adverse scenarios.
